# Best Molecular Tools to Investigate Coronavirus Diversity in Mammals: A Comparison

**DOI:** 10.3390/v13101975

**Published:** 2021-10-01

**Authors:** Petra Drzewnioková, Francesca Festa, Valentina Panzarin, Davide Lelli, Ana Moreno, Barbara Zecchin, Paola De Benedictis, Stefania Leopardi

**Affiliations:** 1 Laboratory of Emerging Viral Zoonoses, Research and Innovation Department, Istituto Zooprofilattico Sperimentale delle Venezie, 35020 Legnaro, Italy; pdrzewniokova@izsvenezie.it (P.D.); ffesta@izsvenezie.it (F.F.); bazecchin@izsvenezie.it (B.Z.); pdebenedictis@izsvenezie.it (P.D.B.); 2 Innovative Virology Laboratory, Research and Innovation Department, Istituto Zooprofilattico Sperimentale delle Venezie, 35020 Legnaro, Italy; vpanzarin@izsvenezie.it; 3 Virology Unit, Istituto Zooprofilattico Sperimentale della Lombardia e dell’Emilia Romagna, 25124 Brescia, Italy; davide.lelli@izsler.it (D.L.); anamaria.morenomartin@izsler.it (A.M.)

**Keywords:** coronavirus, surveillance, pan-CoV, SARS-CoV-2, CaCoV, BoCoV, RT-PCR

## Abstract

Coronaviruses (CoVs) are widespread and highly diversified in wildlife and domestic mammals and can emerge as zoonotic or epizootic pathogens and consequently host shift from these reservoirs, highlighting the importance of veterinary surveillance. All genera can be found in mammals, with α and β showing the highest frequency and diversification. The aims of this study were to review the literature for features of CoV surveillance in animals, to test widely used molecular protocols, and to identify the most effective one in terms of spectrum and sensitivity. We combined a literature review with analyses in silico and in vitro using viral strains and archive field samples. We found that most protocols defined as pan-coronavirus are strongly biased towards α- and β-CoVs and show medium-low sensitivity. The best results were observed using our new protocol, showing LoD 100 PFU/mL for SARS-CoV-2, 50 TCID_50_/mL for CaCoV, 0.39 TCID_50_/mL for BoCoV, and 9 ± 1 log2 ×10^−5^ HA for IBV. The protocol successfully confirmed the positivity for a broad range of CoVs in 30/30 field samples. Our study points out that pan-CoV surveillance in mammals could be strongly improved in sensitivity and spectrum and propose the application of a new RT-PCR assay, which is able to detect CoVs from all four genera, with an optimal sensitivity for α-, β-, and γ-.

## 1. How Diagnostic Failure Can Affect Animal Surveillance

In recent times, coronaviruses (CoVs) have proved to be a major issue for both public and animal health. Indeed, their large genome size, mutation rate, and frequency of recombination seem to make these viruses more susceptible to cross-species transmission and to the subsequent adaptation to new hosts and ecological niches [1,2,3,4,5]. In addition, the shedding of these viruses via fecal and respiratory routes permits easier transmission both between and within host species compared to other agents that require contact with body fluids, such as Ebola, resulting in easier spillover and higher contagiousness.

Coronaviruses are enveloped, positive-stranded RNA viruses that infect mammals and birds. Currently, the subfamily *Orthocoronaviririnae* includes four genera, namely *Alpha-, Beta-, Gamma*-, and *Delta-coronavirus* (α-, β-, γ-, δ-CoV). There are seven CoVs that are known to infect (or have infected) humans. Of these, three emerged through large epidemics climaxing in the ongoing pandemic of COVID-19, caused by the β-CoV severe acute respiratory syndrome coronavirus 2 (SARS-CoV-2) [6,7,8]. Interestingly, human CoVs are all phylogenetically related with viruses found in livestock, especially bovines and camelids, and in wildlife, especially in bats [9,10,11,12,13,14]. This further underscores the need for a deeper understanding of the ecology, evolution, and cross-species transmission of coronaviruses from animal reservoirs.

Before 2003, veterinary surveillance was largely biased towards viruses that resulted in production losses or reduced animal welfare, so that the vast majority of known coronaviruses were associated with domestic animals, including several species of livestock and pets [15,16,17,18]. This picture changed in the aftermath of the human epidemic of severe acute respiratory syndrome (SARS), when we first appreciated the dramatic consequences of CoVs’ emergence in the human population after spilling over from the wild reservoir [19]. Starting from 2005, describing CoV diversity in animals and identifying all its potential reservoir species became a critical step for pandemic preparedness [9,20,21].

Similarly to humans, coronaviruses might emerge in livestock after a cross-species transmission from wildlife. As an example, the highly lethal swine acute diarrhea syndrome (SADS-CoV), which emerged in China between 2016 and 2017, is 96 to 98% similar to the HKU2 virus that is associated with horseshoe bats in the country, thus suggesting it probably originated from this reservoir host [22]. Early detection of emerging CoVs in livestock is critical for both animal and public health. Indeed, even when the spillover of CoVs is not associated with severe disease in the new domestic species, this might maintain and amplify the new virus, acting as a bridge and/or as a reservoir for future human emergence. This seems to have been the case for Middle East respiratory syndrome coronavirus (MERS-CoV), whose animal reservoir is the dromedary camel where the virus most likely spilled from bats, at least 50 years ago [23]. Critically, most CoVs tend to have a similar clinical presentation in livestock, so that the description of an emerging species can be easily delayed or missed in areas endemic for known CoVs if broad-spectrum tests are not implemented, at least in parallel with species-specific tests [24]. For instance, SADS-CoV was initially misdiagnosed as porcine epidemic diarrhea virus (PEDV), another epidemic virus causing similar symptoms in swine and that had caused prior outbreaks at the index farm. As more farms and individuals were involved with a high mortality rate regardless of the control of PEDV, SADS-CoVs was finally diagnosed by metagenomics analysis and retrospective RT-PCR analysis revealed the virus had already present on the index farm during the PEDV epidemic [22].

In this scenario, the development of molecular methods able to detect the greatest variety of CoVs from animals is a critical initial step to ensure early identification. Although next-generation sequencing (NGS) methods are surely the most promising approach for an unbiased discovery of known and (re)emerging pathogens, the implementation of this methodology for virus surveillance is costly and often unsuccessful in field samples with low viral loads compared to classical methods based on the amplification of target genes [25,26]. Similarly, the use of third-generation sequencing approaches, such as the Oxford Nanopore Technologies (MinION), is predicted to become more and more used for the investigation of coronaviruses both in the laboratory and in the field, even if most laboratories still lack the technology, the know-how, or effective protocols. On the other hand, it is critical that PCR-based assays target conserved regions to secure (i) the higher inclusivity for CoVs and (ii) an easy identification by comparison with reference sequences available in public databases, such as the RNA-dependent RNA polymerase (RdRp) [19,27].

Up to now, major attention has been posed to the genera *Alphacoronavirus* and *Beta-coronavirus*, including CoVs with the highest impact on both human and animal health. However, after the identification of *Porcine Deltacoronavirus* (PDCoV) as being a relevant emerging pathogen for the swine industry [28,29], there is now growing evidence that δ-CoVs and γ-CoVs are able to infect and cause disease in mammals as well [30,31]. In this context, it is crucial to mention how previously established panCoV RT-PCR assays have failed to detect the newly emerged PDCoV in diarrheic pig feces subsequently found to be positive using probe-based assays [32]. Thus, as underlined elsewhere [33], there is a critical need to improve available pan-coronavirus methods for the survey of these viruses that might be currently vastly under-detected in mammals.

**Figure 1:**
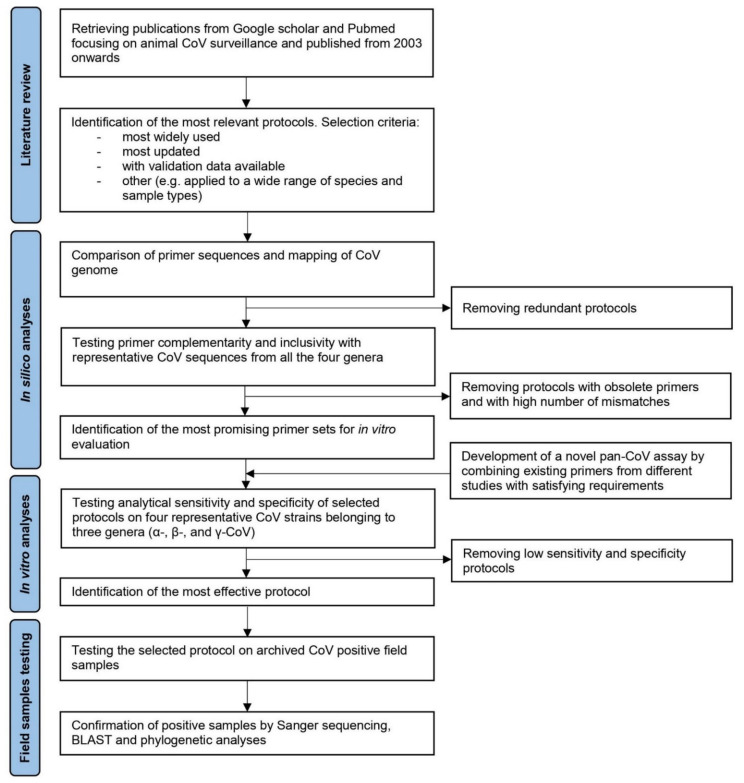
Flow chart for selecting the most suitable molecular protocol for pan-CoV surveillance.

In this study, we critically reviewed the literature on CoV animal surveillance based on molecular tests, to identify the assays currently available for the identification of the four coronavirus genera. Metadata on the target animal species, sampling strategies, and sample matrices adopted were collected and systematically analyzed to selected candidate assays for in silico and in vitro analysis, to ultimately identify the most effective one in terms of analytical sensitivity, specificity, and applicability under field conditions. The selection process of the more suitable protocol for CoV surveillance is shown in Figure 1.

## 2. Literature Review

Google scholar and Pubmed were searched to retrieve a large dataset of scientific literature describing the surveillance of animals for coronaviruses using a broad-spectrum RT-PCR method, including both wildlife and domestic species. In total, 100 papers published between 2003 and 2021 were retrieved. As mentioned, pre-SARS papers (published before 2003) mostly regarded the investigation of specific CoVs infecting livestock, and no attention was posed for the spectrum of the protocols used. For all the papers, we recorded the target species that were assigned to the following categories: “domestic animals”, “bats”, “rodents”, “other wildlife”, and “birds”. In addition, we distinguished between “environmental sampling”, “live sampling”, “passive surveillance”, and “active euthanasia of animals for diagnostic purposes”. We also recorded what samples had been analyzed and their preservation method upon sampling (i.e., the use of lysis buffers or viral transport medium). Results are summarized in Table 1, showing different sampling efforts depending on the animal type, with 71% of publications reporting the screening of bats. The category of domestic animals was the second one in terms of frequency (13%), while fewer studies included the screening of birds (7%). Studies on bat CoVs have been published every year since 2005, while works investigating other animals are sporadic. Studies on domestic animals mostly target single species of interest, especially swine and dromedary camels; on the other hand, studies on wildlife, including bats, rodents, birds, and other mammals, mostly involved different species. Most studies rely on the live sampling of animals. Around 30% of studies also include the screening of carcasses obtained through passive surveillance or from animals euthanized for diagnostic purposes. In particular, 45% and 21% of studies regarding rodents and bats respectively adopted euthanasia. For bats and rodents only, approximately 9% of studies were performed on environmental samples. Most studies preferred the use of gastrointestinal samples (either swabs or faces) for virological screening regardless of the target species. Respiratory samples (mostly oral swabs) were also analyzed in 41% of papers (ranging from 36% and 58% depending on the species), while 28% of the studies included the analysis of organs (ranging from 14% to 64% depending on the species). Nineteen papers reported that when both samples were used, only gastrointestinal matrixes provided positive results; one, referred to bovines, reported positive findings in the respiratory tract only while two reported concordance between respiratory and gastrointestinal samples. Most studies reported the use of field stabilizers: most authors used different viral transport medium, but RNAlater™ (Invitrogen, Massachusetts, USA) or lysis buffers were also employed.

Among the 100 studies analyzed, 52 publications used broad-spectrum primers published in Woo et al./Poon et al. [34,35] (26%) or in De Souza Luna et al. [36] (26%). Other frequently used primers included the ones developed by Chu et al. [37] and Quan et al. [38], referenced in 8% and 6% of papers, respectively (Table 2). Another 48 protocols were used in less than three studies (< 3%). Most of the protocols were successful in the amplification of CoVs belonging to the genera α-CoV and β-CoV, while the genera δ-CoV and γ-CoV were identified only using primers from Chu et al. [37] and a few other methods that were specifically designed for the surveillance of birds [30,39,40,41,42] (Table 2).

## 3. In Silico Evaluation

**Figure 2:**
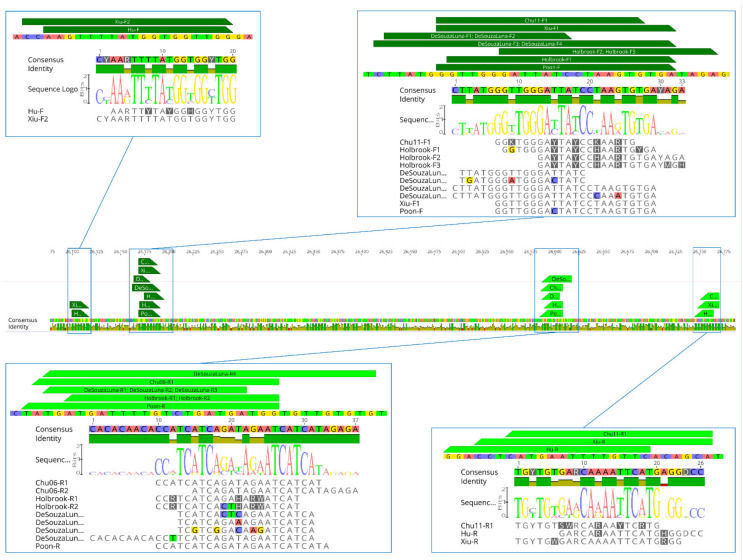
Similarity between primers and their position on the consensus sequence from nucleotide alignment of 69 CoV sequences. Figures were created using Geneious Prime® 2020.1.2 (Biomatters, Auckland, New Zealand).

We selected seven pan-coronavirus protocols, the primers of which were aligned to compare their nucleotide sequences to map their position in the CoV genome. Primer sets selected for analyses in silico included the three most widely used protocols in surveillance studies [34,35,36,37], primers previously published by Chu et al. [43] as well as three updated protocols developed on the basis of recent CoV sequences [32,44,45]. Preliminary analyses showed that most primers are mapped within the same regions of RdRp, and show redundancy in their nucleotide sequences (Figure 2).

For in silico evaluation, we retrieved 69 sequences from the National Center for Biotechnology Information (NCBI) GenBank database, including the reference sequences of each species recognized by the International Committee on Taxonomy of Viruses (ICTV) [46] as well as additional strains of CoVs species reported from different hosts. For each of the four genera, we built a nucleotide alignment using Clustal Omega implemented in Geneious Prime^®^ 2020.1.2 (Biomatters, Auckland, New Zealand) and we assessed the primer–template complementarity, which is crucial for specific amplification. All assays were analyzed for the number and the position of mismatches and tested using Geneious Prime^®^ 2020.1.2, allowing up to 4 mismatches in the binding region of each primer and no mismatches within the last 3 bp of the primer 3′ end, which is assumed to have a significantly larger impact on priming efficiency [47]. Primers that did not meet these criteria were underlined (≥ 5 mismatches) and/or marked (mismatches within 3 bp of primer 3′ end) (Tables S1–S3). Many mismatches (and often towards the 3′ end of the primers) were observed between several tested primers and δ- and γ-CoV sequences (Table 3 and Table S3). Interestingly, primer sets designed by De Souza Luna et al. [37] had a generally low primer–template complementarity (up to 9 mismatches) despite its extensive use in the literature. On the other hand, in silico analyses revealed the best primer–template complementarity between CoV sequences from all four genera and the primers sets Hu-F2/ Hu-R1 and Chu11-F1/Chu11-R1 [32,37]. Detailed results of the complementarity between primers and CoV sequences are presented in Tables S1–S3.

Moreover, based on the in silico results, we set up a novel pan-CoV assay developed by a combination of existing primers from different studies [32,34,35,37]. The complete list of primers tested in silico is presented in Table 3.

## 4. In Vitro Evaluation

For in vitro comparison, we selected the two most promising primer sets, based on their low number of mismatches (i.e., Hu et al. and Chu et al. [32,37]) as well as the oligonucleotides set from De Souza Luna et al. [37] because of its wide application despite their unsatisfactory results in silico (shown in bold red in Table 3). In addition, we tested a new oligonucleotide combination using Hu-F2/ Hu-R1 for the first round of one-step RT-PCR (668 bp), followed by nested PCR with the primers Poon-F and Chu06-R1 (440 bp), to increase assay sensitivity [32,35,43].

We tested all four selected protocols both as first-round amplification and as intended in the original studies in the case of nested approaches. We used the QIAGEN OneStep RT-PCR kit (QIAGEN, Hilden, Germany) for the first round of all RT-PCR assays, and the Platinum™ Taq DNA Polymerase (Invitrogen, MA, USA) for the second round of the assays, as indicated in the original studies [32,36]. Primer concentration and thermal cycles were adopted as indicated in the original studies [32,36]. Whenever exact protocols were not available, we followed the recommendations provided by the manufacturers in the manual of each amplification kit [37]. Further details are described in the Supplementary Materials Figure S1.

The analytical sensitivity of single assays was evaluated for three CoV genera using cell-adapted viral strains, namely one α-CoV (canine coronavirus, CaCoV), two β-CoVs (bovine coronavirus, BoCoV and SARS-CoV-2) and one γ-CoV (infectious bronchitis virus, IBV) (Table 4). Unfortunately, no isolates of δ-CoVs were available for this test.

For each virus, we obtained 10-fold serial dilutions and extracted the RNA using the QIAamp^®^ Viral RNA Mini kit (QIAGEN, Hilden, Germany) according to the manufacturer’s instructions. All analyses were run in triplicate for each dilution. For SARS-CoV-2 only, we compared the sensitivity of each method with the specific real-time RT-PCR targeting E gene widely used for the diagnosis of infection in humans and animals [48].

**Figure 3:**
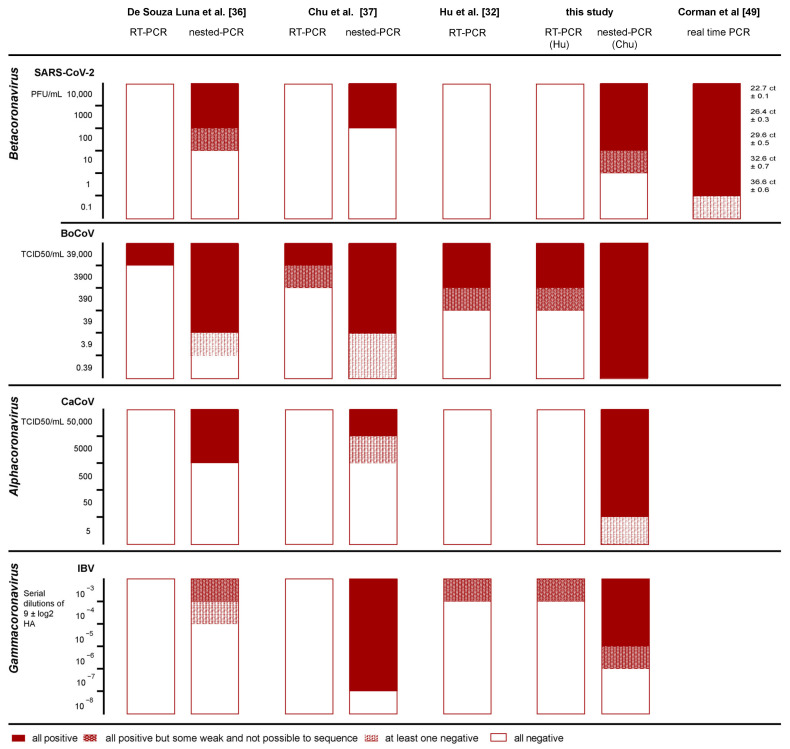
In vitro evaluation of primer specificity and comparison of protocols’ sensitivity. Red blocks represent serial dilution that provided positive results in all repetitions. The higher dilution at which all the replicas yielded positive results identified the LOD of the test. Red/white patterns show cases in which even lower dilution resulted in weak positivity or inconsistent results among the different repetitions. † Antigen suspension with initial titer 9 ± 1 log2 HA.

All protocols revealed unsatisfactory results in the first round, failing to detect SARS-CoV-2 and CaCoV even at high concentrations (1 × 10^4^ PFU/mL and 5 × 10^4^ TCID50/mL, respectively). Among the nested PCRs, the highest sensitivity for α- and β-CoVs was observed with the newly designed protocol, showing a limit of detection (LoD) of 100 PFU/mL SARS-CoV-2, 50 TCID_50_/mL CaCoV, and 0.39 TCID_50_/mL BoCoV. For IBV, the highest sensitivity (5th 10-fold dilution of 9 ± 1 log2 HA) was observed employing the primer set published by Chu et al. [37]. As expected, species-specific real-time RT-PCR was the most sensitive assay for SARS-CoV-2 detection. Further details are shown in Figure 3.

## 5. Field Evaluation

In order to test the performances of the new nested assay optimized in this study for animal surveillance, we analyzed 30 field archive samples representative of different field conditions and all four genera of coronavirus. In particular, we selected different samples originating from a wide variety of wild and domestic animals that were previously confirmed as positive for CoVs using different approaches, including other pan-coronavirus methods [36,37], species-specific PCRs, and NGS. We tested different kind of sample matrixes among the ones that are mostly used in animal surveillance, including feces, anal swabs, saliva, salivary swabs, and organs (pools and intestines). Samples were collected up to 8 years ago and stored either dry or using different field stabilizers (Table S4). For RNA extraction, we used either the QIAamp^®^ Viral RNA Mini kit (QIAGEN, Hilden, Germany), NucleoSpin RNA Mini kit (MACHEREY-NAGEL, Düren, Germany), or MagMAX Pathogen™ RNA/DNA Nucleic Acid Kit (Applied Biosystems, MA, USA).

The new protocol was able to confirm the presence of CoV RNA in all the archived samples, including the different matrixes that are commonly used for CoV detection and regardless of the collection strategies and the age of samples (Table S4). Positive samples were confirmed through Sanger sequencing, which provided clean sequences of about 440 base pairs. Nucleotide sequences were analyzed trough the BLAST online tool (Rockville Oike, USA) 

**Figure 4:**
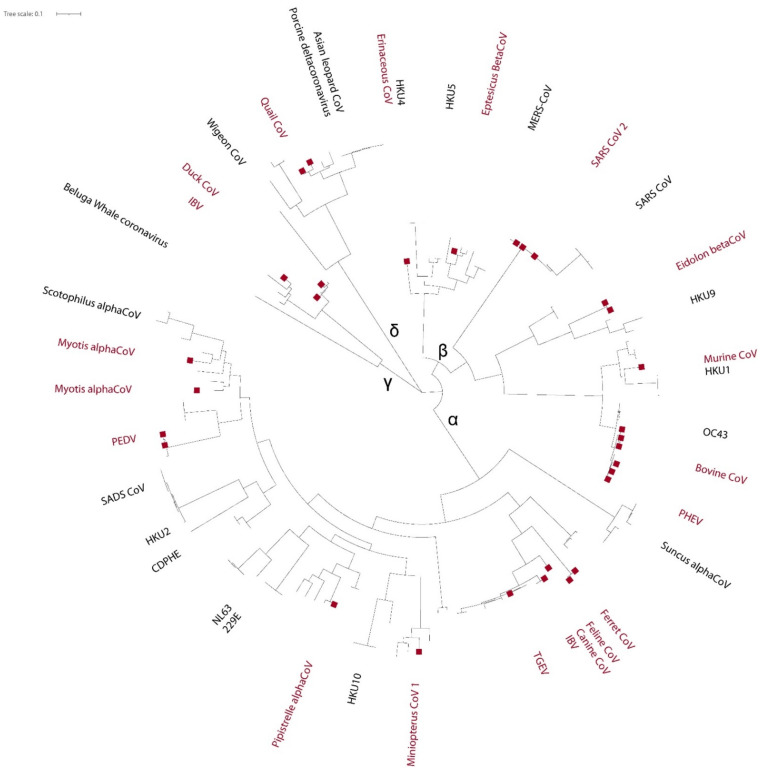
ML phylogenetic tree of coronaviruses. Sequences were aligned using the G-INS1 and default parameters implemented in Mafft using online tool: https://mafft.cbrc.jp/alignment/server [50]. ML phylogenetic trees were inferred using PhyML (version 3.0) implemented in Seaview (Lyon, France), employing the GTR+G4 substitution model, a heuristic SPR branch-swapping algorithm, and SH-like branch supports [51]; obtained trees were edited online for graphical display using iTOL on server: https://itol.embl.de/ [52]. CoV species investigated in the study are indicated in red, with branches of tested strains shown as red boxes.

For primary identification purposes, we aligned the nucleotide sequences of all tested strains together with the reference sequences retrieved from GenBank and representative of the CoV diversity found in animals and built a maximum likelihood (ML) phylogenetic tree. Detected strains belonged to all CoV genera and were isolated from several hosts, including swab/salivary samples positive for SARS-CoV-2 showing *Ct* values up to 30.76 (Figure 4, Table S4). For all classified CoVs, this analysis allowed correct identification, confirming the specificity of the newly developed test. Besides, phylogenetic analysis of the sequences obtained was sufficiently informative to allow classification within known subgenera of all CoV strains that do not meet parameters for official classification (Figure 4) [49].

## 6. A Look into the Future of Coronavirus Surveillance

The ongoing pandemic of COVID-19 shows the dramatic consequences that emergent coronaviruses may have on a naïve population. Similar to what has been seen in humans, novel CoVs can infect livestock, causing epidemics that may evolve into large epizootics, and causing severe economic consequences, as shown by the worldwide spread of porcine epidemic diarrhea virus (PEDV) [53]. In both humans and livestock, coronaviruses emerge after spilling over from a reservoir host that maintains coronaviruses in nature [4]. In this view, the screening of wildlife is providing increasing information about the large diversity of this viral family in a wide variety of animals, but with the highest frequency and diversification in the order *Chiroptera*, namely in bats. However, we found that the literature is largely skewed towards the investigation of these animals, where SARS-like viruses were first found [54], and this may generate confounding data. Indeed, it is increasingly clear that we could find a similar diversity of CoVs in other animals as well, if we searched more robustly, such as in rodents and birds [9,10,31,55,56]. In this context, the few CoV species described in rodents, which make up approximately 40% of all mammalian species, seem too much in contrast with the large diversity of CoVs found in bats, and this might be explained by the lower sampling effort and the limited number of target species, as shown in our selected literature review.

Another interesting point that emerged from the analyzed literature that refers to pan-CoV surveillance is the fact that fewer than 41% of the studies tested the respiratory tract of animals, compared to almost 80% of papers describing the testing of feces and/or rectal swabs. This approach is due to the higher probability for feces to test positive [9]. However, we note that this consideration may differ in different species. Indeed, while CoVs in bats seem to have mostly a gastroenteric tropism, most human viruses and some of the CoV species described in companion and domestic animals are associated with the respiratory tract. Fortunately, several studies have confirmed that these viruses can also be found in feces if a molecular approach is applied, and they also encourage the use of this matrix in case of limited resources or if other sample types cannot be processed or collected [9]. However, it is likely that the parallel use of oral/nasal swabs would increase the sensitivity of unbiased surveillance.

Currently, all data suggest that alpha- and beta-coronaviruses have evolved in mammalian hosts while birds are the evolutionary reservoir for gamma- and delta-coronaviruses [40]. However, new coronaviruses that have been recently described in mammals include a divergent gamma-coronavirus in a captive beluga whale (*Delphinapterus leucas*) [30] and new delta-coronaviruses in Asian leopard cats *(Prionailurus bengalensis*) and in Chinese ferret badgers (*Melogale moschate*) found in wet markets. In addition, PDCoV is rapidly emerging in the swine industry in both the USA and China, leading to a severe disease with consequent economic losses [57]. Overall, these data suggest that the circulation of delta and gamma-coronaviruses in mammals might be underestimated [31]. Indeed, our results emphasize how most of the protocols widely used in mammals actually fail to detect γ- and δ-CoVs, suggesting that data might be confounded by methodological constrains, leading to a substantial under-sampling of mammals for these viruses [31]. In particular, most of the protocols reported as pan-coronavirus were not actually tested for γ- and δ-CoVs [36,38], and often included primers with low complementarity against their target regions and several mismatches located within their 3′ end. For γ-CoVs, these data were confirmed in vitro, with most protocols showing low sensitivity. While it was not possible to perform similar analyses for δ-CoVs, the consistency obtained between the analyses performed in silico and in vitro for the other three genera confirms how primer–template complementarity strongly influences the success of PCRs and suggests that analyses in silico can be used as a good proxy when isolates are unavailable for actual testing.

We observed the worst results using the primer sets published by De Souza Luna et al. [36], which showed a high number of mismatches with γ- and δ- CoVs and very low performances for the amplification of IBV (γ-CoV), failing to detect all tree replicates even at the highest concentration. It is worth noting that this protocol is still one of the most widely used in the surveillance for CoVs despite being one of the first ones to be developed using the alignment of the few viral sequences available at that time. We obtained similar unsatisfying results for most widespread protocols, with an exception made for the one designed by Chu et al. [37], which was actually intended for surveillance in birds. In addition, as our results demonstrate, the sensitivity of these protocols was low even for α- and β-CoVs.

Technically speaking, all protocols considered in this study included primers overlapping with each other in the same portions of the RNA-dependent RNA polymerases (*RdRp*). This choice is related to the fact that this gene is highly conserved among different coronaviruses and provides sequences of sufficient length for phylogenetic studies and a preliminary classification [27]. In addition, the amplification of the same fragment secures an easier comparison among CoVs using different assays. However, the protocols analyzed in this study differ in the number and position of degenerations, thus affecting the range of CoV species with sufficient complementarity. For example, primers by De Souza Luna et al. [36] and by Poon et al. [35] contain no degenerations and show very low primer and template complementarity with CoVs divergent from the ones used for the development of the assay, especially for the reverse primer. Since the start of the COVID-19 pandemic, few novel pan-CoVs assays have been published [44,45], but the complementarity of their primer sets with most CoVs is only moderate. On the other hand, primers used by Chu et al. in the first round include relevant degenerations that improved the performances of this assay both in silico and in vitro [37]. A nested approach using non-degenerated primers for the second amplification round was implemented. Actually, the use of nested or hemi-nested protocols is well established and used by most authors. This choice is well supported by our data, which show that all the protocols provide highly unsatisfying results for all the analyzed viruses in the first round. On the other hand, we showed how nested PCRs significantly increased the sensitivity of all the assays, in some cases up to five logarithms. This is in agreement with results from another study, where the authors observed a difference of five to eight logarithms between the first and second step of the pan-CoV assay carried out on serial dilutions of SARS-CoV-2 and MERS-CoV [45]. Nested PCR may be omitted only in case of a high viral load in the samples, although field samples (especially swabs) are likely to have low concentrations of viral RNA. This was demonstrated in a study analyzing anal swabs of bats, where 389 copies of SARS-CoV RNA/mL [54] were detected. Similarly, in another study, samples of different origins (rectal, nasal, and ocular swabs) tested for the presence of BoCoV RNA showed concentrations varying from 8.0 × 10^8^ to 2.2 × 10^1^ RNA/µL [58]. Therefore, we suggest that using only the first step of RT-PCRs for field surveillance may easily lead to false negative results. We confirmed this hypothesis on field samples analyzed using our novel approach that turned out positive after the second step of amplification. Among the different protocols described in the literature, the one developed by Hu et al. is one of the few relying on a single step of amplification [32]. As expected, this assay showed the lowest sensitivity overall for all CoVs under investigation. Nevertheless, its degenerated primers showed an excellent complementarity with coronaviruses, revealing zero (FW: *n* = 62; REV: *n* = 41) or at maximum one (FW: *n* = 7; REV: *n* = 27) mismatch with most CoVs analyzed (*n* = 69), with the only exception being *Beluga whale coronavirus*, accounting for two mismatches only, one of which is located toward the 3′ end of the primer. This is consistent with our results from in vitro analyses, which showed a higher sensitivity of this protocol compared to the first rounds of all other protocols tested. Thus, we decided to combine these primers with nested primers from Chu et al.with the aim of developing a new approach for the detection of CoV RNA from all four genera (α-, β-, γ-, and δ- CoV) while securing higher sensitivity compared to the published assays [37].

Such results confirmed that our new assay showed an increased sensitivity for all α- and β-CoV in vitro, but in the case of IBV (γ-CoV), better sensitivity was observed with primers from Chu et al., whose aim was to identify CoVs in bird samples [37]. Notably, we were able to detect as little as 10 PFU/mL of SARS-CoV-2, even if 100 PFU/mL was established as a limit of detection because some of the replicates of the highest dilution were weak and sequencing was unsuccessful. Compared to the broadly used species-specific rRT-PCR targeting the *E* gene [48], our protocol resulted in the loss of approximately two logarithms and false negatives for dilutions showing 36 *Ct* through rRT-PCR. However, our data from field samples suggest this test could be sensitive enough for its application in the field, just as we were successful in confirming positivity for all the archived samples belonging to all four CoV genera. Indeed, our protocol successfully identified all positive field samples, which included different matrixes, hosts, and viruses, showing that its application in surveillance programs is promising for both wildlife and domestic animals. This evidence is crucial because the used of broad-spectrum approaches is frequently avoided in veterinary surveillance, especially in case of disease outbreaks, in favor of probe-based protocols. Indeed, our study demonstrated how unbiased surveillance of coronaviruses is still very low in domestic animals. This is mostly due to the fact that probe-based molecular methods are faster, more sensitive, and scalable compared to broad-spectrum protocols, which often require a nested or hemi-nested approach to secure acceptable sensitivity. However, targeted analyses might confound diagnosis during epidemics, especially because coronaviruses often cause similar symptoms. In addition, we recently found that even epidemic viruses, such as PEDV, could circulate endemically in pig herds in the absence of symptoms [24]. This implies that specific PCRs might turn out to be positive even when the targeted pathogen is not responsible for the clinical disease, potentially delaying the detection of novel viruses, as seen during the emergence of SADS-CoV [22]. Of note, the case of MERS shows how the emergence of coronaviruses in the human population might be favored by a previous passage in domestic animals, which can both amplify or modify the viruses, increasing either infectivity, transmissibility, or pathogenicity for humans. In this context, broad-spectrum surveillance in livestock is of utmost importance not only in terms of animal health per se, but also in terms of public health and conservation, as it allows early detection of a spill-over event. The use of our pan-coronavirus approach might combine the chances of detecting known and unknown coronaviruses from all matrixes that are commonly used in animal surveillance with an acceptable sensitivity, thus overcoming costly and time-consuming approaches, such as metagenomics or the application of several targeted protocols. In addition, our method allows a preliminary classification of the infectious agent by sequencing and phylogenetic analysis, leading to the identification of circulating or emerging coronaviruses. As demonstrated by Chan et al., pan-CoV amplification can be combined with the MinION sequencer [59]. Therefore, further research should focus on the implementation of portable next-generation sequencers to provide rapid and cost-effective preliminary classification.

## Figures and Tables

**Table 1:** Results for the literature review, indicated as number of papers screened that related to a particular category in the table (percentage).

Host	*N*	Targeted animal species		Sampling strategy *					Matrix		
		Single species	multi species	L	E	P	S	field stabilizer	feces/ anal swab	oral swab	organs
Bats	71	13 (18)	58 (82)	55 (77)	7 (10)	4 (6)	15 (21)	39 (55)	61 (86)	30 (42)	19 (27)
Rodents	11	0	11 (100)	7 (64)	1 (9)	1 (9)	5 (45)	8 (73)	7 (64)	4 (36)	7 (64)
Other wild mammals	12	5 (41)	8 (67)	10 (83)	0	2 (17)	2 (17)	10 (83)	9 (75)	7 (58)	4 (33)
Domestic mammals	13	10 (77)	3 (23)	11 (85)	0	3 (23)	0	1 (7.7)	8 (62)	6 (46)	2 (15)
Birds	7	0	7 (100)	7 (100)	0	0	1 (14)	6 (86)	7 (100)	4 (57)	1 (14)
Total	100	27	74	77	8	9	21	54	79	41	28

*N* = number of papers. In the section, “targeted animal species” is indicated if the study describes the targeted sampling of a single species or if more than one species is tested due to opportunistic sampling (such as trapping or netting). * Acronyms for the sampling strategies refer to live sampling (L), environmental sampling (E), passive surveillance (P), and sacrifice of animals (S).

**Table 2:** Pan-CoV protocols mostly used in the literature according to the host, and the relative success rate in the identification (id) of coronaviruses from the four genera.

Host	*N*	Woo et al./Poon et al. [34,35]	De Souza Luna et al. [36]	Chu et al. 2011 [37]	Quan et al. [38]	Others (< 3 papers)
Bats	71	19 (27%)	22 (31%)	2 (3%)	5 (7%)	29 (41%)
Rodents	11	4 (36%)	4 (36%)	0	3 (27%)	4 (36%)
Other wild mammals	12	5 (42%)	3 (25%)	1 (8%)	3 (25%)	4 (33%)
Domestic mammals	13	2 (15%)	2 (15%)	4 (31%)	0	5 (38%)
Birds	7	1 (14%)	0	1 (14%)	0	5 (71%)
Total	100	26%	26%	8%	6%	42%
Identification of α-cov		18 (69%)	19 (73%)	3 (38%)	3 (50%)	25 (60%)
Identification of β-cov		20 (17%)	21 (81%)	6 (75%)	6 (100%)	23 (55%)
Identification of γ-cov				1 (13%)		3 (7%)
Identification of δ-cov				1 (13%)		3 (7%)

**Table 3:** Pan-CoV primers selected for in silico analyses. Primer names are shown as the author’s name for easier recognition. Data were generated by primer3 in Geneious Prime^®^ 2020.1.2 (Biomatters, Auckland, New Zealand).

Ref.	PCR format	Primer	S/As	Sequence (5′→3′)	Deg. *	Number of Mismatches			
						α	β	γ	δ
[44]	Two-step RT-	Holbrook-F1	S	GGTGGGAYTAYCCHAARTGYGA	48	0–2	1–4	0–1	0–1
		Holbrook-R1	As	CCRTCATCAGAHARWATCAT	24	0–3	0–3	1–3	0–1
		Holbrook-R2	As	CCRTCATCACTHARWATCAT	24	0–5	0–5	1–4	2–3
	Hemi–nested	Holbrook-F2	S	GAYTAYCCHAARTGTGAYAGA	48	0–3	0–3	0–1	1–2
		Holbrook-F3	S	GAYTAYCCHAARTGTGAYMGH	288	0–1	0–4	0–1	0–1
[45]	One-step RT-	Xiu-F1	S	CYAARTTTTATGGTGGYTGG	8	0–2	0–4	0–2	0–1
		Xiu-R	As	TGYTGWGARCAAAATTCATGRGG	16	0–3	0–3	0–2	0–2
	Hemi-nested	Xiu-F2	S	GGTTGGGATTATCCTAAGTGTGA	None	1–4	0–4	0–3	0–3
[32]	**One-step RT-**	**Hu-F**	**S**	**AARTTYTAYGGHGGYTGG**	**48**	**0–1**	**0–1**	**0–1**	**0**
		**Hu-R**	**As**	**GARCARAATTCATGHGGDCC**	**36**	**0–1**	**0–1**	**0–2**	**0–1**
[37]	**Two-step RT-**	**Chu11-F1**	**S**	**GGKTGGGAYTAYCCKAARTG**	**32**	**0–2**	**0–1**	**0–1**	**0–2**
		**Chu11-R1**	**As**	**TGYTGTSWRCARAAYTCRTG**	**128**	**0–1**	**0–1**	**0–1**	**0–1**
	Nested	Chu11-F2	S	*Identical to Poon-F*					
		Chu11-R2	As	*Identical to Chu06-R1*					
[43]	Two-step RT-	Chu06-F1	S	*Identical to Poon-F*					
		**Chu06-R1**	**As**	**CCATCATCAGATAGAATCATCAT**	**None**	**1–6**	**2–5**	**4–8**	**2–5**
		Chu06-F2	S	*Identical to Poon-F*					
		Chu06-R2	As	ATCAGATAGAATCATCATAGAGA	None	1–7	2–8	5–11	5–9
[36]	**One-step RT-**	**DeSouzaLuna-F1**	**S**	**TTATGGGTTGGGATTATC**	**None**	**0–4**	**0–3**	**0–3**	**3–6**
		**DeSouzaLuna-F2**	**S**	**TGATGGGATGGGACTATC**	**None**	**0–4**	**1–4**	**1–3**	**4–7**
		**DeSouzaLuna-R1**	**As**	**TCATCACTCAGAATCATCA**	**None**	**2–7**	**1–6**	**4–7**	**2–7**
		**DeSouzaLuna-R2**	**As**	**TCATCAGAAAGAATCATCA**	**None**	**0–5**	**0–5**	**3–4**	**1–5**
		**DeSouzaLuna-R3**	**As**	**TCGTCGGACAAGATCATCA**	**None**	**1–7**	**3–7**	**1–5**	**3–5**
	**Nested**	**DeSouzaLuna-F3**	**S**	**CTTATGGGTTGGGATTATCCTAAGTGTGA**	**None**	**1–6**	**0–5**	**0–3**	**3–7**
		**DeSouzaLuna-F4**	**S**	**CTTATGGGTTGGGATTATCCCAAATGTGA**	**None**	**0–7**	**1–5**	**1–5**	**5–9**
		**DeSouzaLuna-R4**	**As**	**CACACAACACCTTCATCAGATAGAATCATCA**	**None**	**2–9**	**4–7**	**5–8**	**4–8**
[34] [35]	Two-step RT-	**Poon-F**	**S**	**GGTTGGGACTATCCTAAGTGTGA**	**None**	**0–4**	**0–3**	**1–2**	**1–4**
		Poon-R	As	CCATCATCAGATAGAATCATCATA	None	1–6	3–5	4–8	3–6
**This study**	**One step RT-**			**Identical to Hu** [32]					
	**Nested**			**Identical to Poon-F and Chu06-R1** [35,43]					

S: sense primers, As: antisense primers. The primers that were further tested in vitro are shown in bold. * Deg.—primer degeneracy (a number of unique sequence combinations). Detailed information about number of mismatches and information about presence of mismatch within the 3′ end of primer is presented in Tables S1–S3.

**Table 4:** Viral isolates used in this study to compare pan-CoV protocols. No δ-CoVs were available to assess analytical sensitivity.

Genus	Species	Strain	Material	Titer
α	Alphacoronavirus 1—Canine CoV	Jan 71 (ATCC VR-809)	cell culture supernatant	5.0 × 10^7^ TCID50/mL
β	Betacoronavirus 1—Bovine CoV	Mebus	cell culture supernatant	3.9 × 10^6^ TCID50/mL
β	SARS-CoV-2	IZSVe20VIR1935	cell culture supernatant	1.0 × 10^5^ PFU/mL
γ	Infectious Bronchitis Virus	D1466	egg allantoic fluid	9 ± 1 log2 HA

## Supplementary Materials

The following are available online at https://www.mdpi.com/article/10.3390/v13101975/s1. Methodological details of in vitro evaluation of selected protocols. Figure S1. Analytical sensitivity of the novel assay developed in this study. Table S1. Complementarity of primers with *Alfacoronavirus* sequences. Table S2. Complementarity of primers with *Betacoronavirus* sequences. Table S3. Complementarity of primers with *Gammacoronavirus* and *Deltacoronavirus* sequences. Table S4. Field samples and viral strains used to test the inclusivity of the novel assay developed in this study.
