# Supplementary material for: Best Molecular Tools to Investigate Coronavirus Diversity in Mammals: A Comparison
Source: Viruses. 2021 Oct 1;13(10):1975. doi: 10.3390/v13101975 (PMC8538982; doi:10.3390/v13101975)
Supplement: Supplementary file 1 [file viruses-13-01975-s001.zip › Figure S1.pdf]

Supplemental material to "Best molecular tools to investigate coronavirus diversity in mammals: a comparison"

Petra Drzewnioková, Francesca Festa, Valentina Panzarin, Davide Lelli, Ana Moreno, Barbara Zecchin, Paola De Benedictis and Stefania Leopardi

In vitro evaluation of selected protocols

The sensitivity of single assays was evaluated for three CoV genera using cell adapted viral strains, namely one α-CoV (Canine coronavirus - CaCoV), two β-CoVs (Bovine coronavirus – BoCoV and SARS-CoV-2) and one γ-CoV (Infectious Bronchitis virus - IBV). Each session included one sample (Feline coronavirus) as positive control, one negative process control and three no template controls (NTC) for the first round of RT-PCR. To perform nested PCR one more NTC was added. We used mineral oil to prevent cross-contaminations between dilutions. Reactions of the first rounds were carried out using the QIAGEN OneStep RT-PCR kit (QIAGEN) according to the manufacturer's instructions. Briefly, reaction mixtures (25 µl) consisted of 1 µl of the enzyme mix, 0.4 mM of each dNTPs, 10U of RNasin® Ribonuclease Inhibitor (Promega) and five microliters of RNA. One microliter of DNA from the first round was used as template in the nested PCR (25-µl mixtures), which was carried out using the Platinum™ Taq DNA Polymerase (Invitrogen) according to the manufacturer's instructions. Primer concentrations and thermal cycles of single PCR protocols are described below. Gel electrophoresis was performed using QIAxcel Advanced System (Qiagen).

<u>De Souza Luna et al., 2007</u> [1]

1° round: RT-PCR (size of PCR product: 441-455 bp):

- *Forward primer mix*: DeSouzaLuna-F1 and DeSouzaLuna-F2 in final concentration 0.2 µM each
- *Reverse primer mix*: DeSouzaLuna-R1, DeSouzaLuna-R2 and DeSouzaLuna-R3 in final concentration 0.9 µM each
- *Thermal conditions*: 30 min at 50°C; 15 min at 95°C; 10 cycles of 20 s at 94°C, 30 s starting at 62°C with a decrease of 1°C per cycle, and 40 s at 72°C; 30 cycles of 20 s at 95°C, 30 s at 52°C, and 40 s at 72°C; and 1 cycle at 72°C for 10 min

2° round: nested PCR (size of PCR product: 454 bp):

- *Forward primer mix*: DeSouzaLuna-F3 and DeSouzaLuna-F4 in final concentration 0.08 µM each
- *Reverse primer*: DeSouzaLuna-R4 in final concentration 0.4 µM
- *Thermal conditions*: 3 min at 94°C; 30 cycles of 20 sec at 94°C, 30 sec at 52°C and 30 sec at 72°C

<u>Chu et al., 2011</u> [2]

1° round: RT- PCR (size of PCR product: 602 bp):

- *Forward primer:* Chu11-F1 in final concentration 0.6 µM
- *Reverse primer:* Chu11-R1 in final concentration 0.6 µM
- *Thermal conditions:* 30 min at 50°C; 15 min at 95°C; 40 cycles of 20 sec at 94°C, 40 sec at 50°C and 1 min at 72°C; and 10 min at 72°C

2° round: nested PCR (size of PCR product: 440 bp):

- *Forward primer:* Chu11-F2 in final concentration 0.4 µM
- *Reverse primer:* Chu11-F2 in final concentration 0.4 µM
- *Thermal conditions:* 3 min at 94°C; 30 cycles of 20 sec at 94°C, 30 sec at 50°C and 50 sec at 72°C

<u>Hu et al., 2018</u> [3]

RT- PCR (size of PCR product: 668 bp):

- *Forward primer*: Hu-F in final concentration 1 µM
- *Reverse primer*: Hu-R in final concentration 1 µM
- *Thermal conditions:* 30 min at 50°C; 15 min at 95°C; 35 cycles of 20 sec at 94°C, 40 sec at 52°C and 1 min at 72°C; and 10 min at 72°C.

<u>This study</u>
1° round: *identical to RT- PCR Hu et al.*, 2018
2° round: *identical to nested PCR Chu et al., 2011*
Results of the analytical sensitivity are presented in figure S1.

References:

1. Luna, L.K. de S.; Heiser, V.; Regamey, N.; Panning, M.; Drexler, J.F.; Mulangu, S.; Poon, L.; Baumgarte, S.; Haijema, B.J.; Kaiser, L.; et al. Generic Detection of Coronaviruses and Differentiation at the Prototype Strain Level by Reverse Transcription-PCR and Nonfluorescent Low-Density Microarray. *Journal of Clinical Microbiology* **2007**, *45*, 1049–1052, doi:10.1128/JCM.02426-06.
2. Chu, D.K.W.; Leung, C.Y.H.; Gilbert, M.; Joyner, P.H.; Ng, E.M.; Tse, T.M.; Guan, Y.; Peiris, J.S.M.; Poon, L.L.M. Avian Coronavirus in Wild Aquatic Birds. *Journal of Virology* **2011**, *85*, 12815–12820, doi:10.1128/JVI.05838-11.
3. Hu, H.; Jung, K.; Wang, Q.; Saif, L.J.; Vlasova, A.N. Development of a One-Step RT-PCR Assay for Detection of Pancoronaviruses (α-, β-, γ-, and δ-Coronaviruses) Using Newly Designed Degenerate Primers for Porcine and Avian `fecal Samples. *Journal of Virological Methods* **2018**, *256*, 116–122, doi:10.1016/j.jviromet.2018.02.021.

Figure S1. Analytical sensitivity of the novel assay developed in this study. The limit of detection is marked in red.

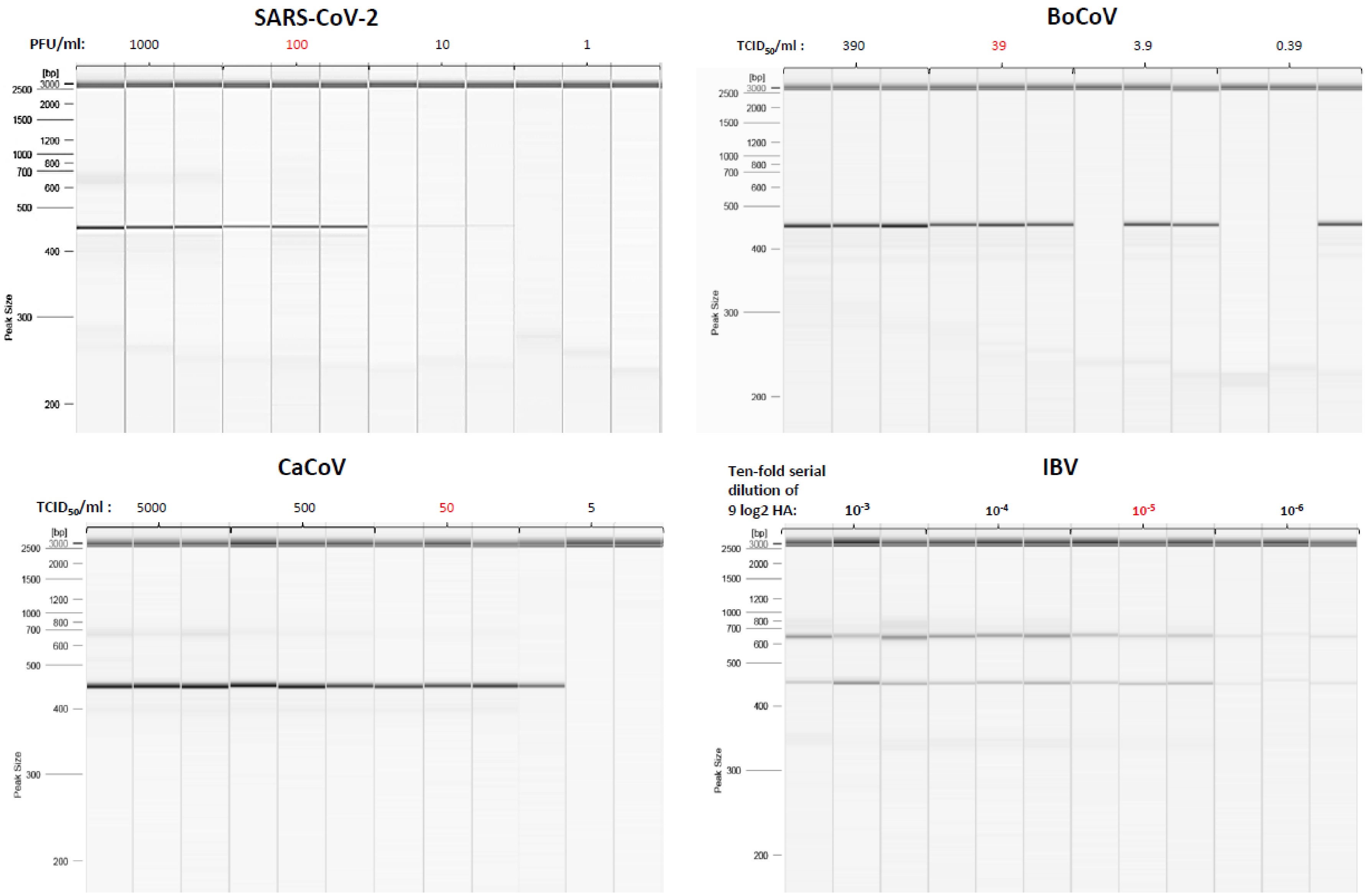

Table S1. Complementarity of primers with *Alfacoronavirus* sequences. The primers that did not bind to the template in *in silico* analyses are underlined (*the mismatches were present within the 3 bp of 3′end of primer). Primer sets where at least one primer had no mismatch with the template sequence are in **bold**.

Species	Subspecies		Isolate	Accession No.	Hu-F	Hu-R	Chu11-F1	Chu11-R1	Chu06-R1	Chu06-R2	Poon-F	Poon-R	DeSouzaLuna-F1+2	DeSouzaLuna-R1+2+3	DeSouzaLuna-F3+4	DeSouzaLuna-R4	Holbrook-F1	Holbrook-R1+2	Holbrook-F2+3	Xiu-F1	Xiu-R	Xiu-F2
Alphacoronavirus 1	*TGEV*		Purdue	AJ271965	**0**	**0**	1	**0**	1	2*	1	1	**3; 0**	4; 2; 4	4; 6	3	1	**0; 2**	**2; 0**	**0**	1	2
	Feline CoV	*Type I*	C1Je	DQ848678	**0**	**0**	1	1	3	4*	1	3	**3; 0**	4; 2; 4	4; 6	3	1	1; 3	**2; 0**	1	2	2
		Type II	FIP virus WSU 79-1146	AY994055	**0**	**0**	**0**	**0**	1	2*	1	1	3; 1	4; 2; 4	4; 6	3	**0**	**0; 2**	**2; 0**	**0**	1	2
	Porcine respiratory CoV		ISU-1	DQ811787	**0**	**0**	1	**0**	1	2*	1	1	**3; 0**	4; 2; 4	3; 5	3	1	**0; 2**	**2; 0**	**0**	1	2
	Canine CoV	*Type I*	23/03	KP849472	**0**	**0**	1	1	1	2*	1	1	**3; 0**	4; 2; 4	4; 6	4	1	**0; 2**	**2; 0**	1	2	2
		Type II	NTU336/F/2008	GQ477367	**0**	**0**	1	**0**	1	2*	1	1	3; 1	4; 2; 4	4; 6	3	1	**0; 2**	**2; 0**	**0**	**0**	2
		Type IIa	CB/05	KP981644	**0**	**0**	1	**0**	2	3*	1	2	**3; 0**	3; 2; 3	4; 6	4	1	**0; 2**	**2; 0**	**0**	1	2
		Type IIb	174/06, type IIb	EU856362	**0**	**0**	1	**0**	2	3*	1	2	**3; 0**	3; 2; 3	4; 6	4	1	**0; 2**	**2; 0**	**0**	1	2
Bat coronavirus HKU10			183A	NC_018871	**0**	1	**0**	**0**	2	3*	1	2	2*; 3*	3; 2; 3	2; 4	5	1	**0; 2**	**0; 0**	**0**	2	2
Bat coronavirus CDPHE15			bat/USA/CDPHE15/2006	NC_022103	**0**	1	**0**	**0**	4	5*	2	4	3; 2	7; 5; 3	4; 5	6	1	2; 4	**0; 0**	**0**	1	3
Human coronavirus 229E			KW2E-F151/Hipcfrub/GHA/11	KT253269	**0**	1	**0**	**0**	3	3*	**0**	3	2; 1	4; 1; 4	3; 5	3	1	1; 3	**0; 0**	**0**	2	1
			Human coronavirus 229E	NC_002645	1	**0**	1	**0**	2	2*	1	2	**3; 0**	**3; 0; 5**	4; 6	3	1	1; 3	**0; 0**	2	**0**	2
			camel/Riyadh/Ry141/15	NC_028752	**0**	**0**	1	**0**	1	2*	1	1	3; 1	**3; 0; 5**	4;6	2	1	**0; 2**	**0; 0**	2	**0**	2
Human coronavirus NL63			NL63 Amsterdam I	AY567487	**0**	1	1	1	2	3*	3	3*	**0; 3**	3; 1; 5	**2; 0**	3	1	1; 3	**0; 0**	**0**	3	2
Lucheng Rn rat coronavirus			Lucheng-19	NC_032730	**0**	**0**	1	**0**	1	2*	1	1	2; 2	4; 2; 4	3; 3	2	1	**0; 2**	**2; 0**	1	**0**	2
Miniopterus bat CoV 1	B		AFCD307	NC_010436	1	1	1	1	3	3	1	3	3; 2	4; 3; 3	4; 6	4	1	1; 3	**0; 0**	2	1	2
	A		AFCD62	NC_010437	1	**0**	1	1	2	2	2	2	2; 2	5; 3; 4	3; 3	4	1	2; 4	**0; 0**	1	**0**	3
Miniopterus bat CoV HKU8			AFCD77/08/05 Mm	NC_010438	**0**	1	**0**	1	2	1	2	2	2*; 4*	2; 1; 4	2; 4	3	1	**0; 2**	**2; 0**	2	1	1
Mink coronavirus 1			WD1127	NC_023760	**0**	**0**	**0**	1	2	2	3	2	0; 3	5; 3; 4	2; 1	5	2	**0; 2**	3; 1	**0**	2	2
Myotis ricketti alphaCoV			Sax-2011	NC_028811	**0**	1	**0**	**0**	6*	7*	**0**	6*	1; 2	6; 5; 2	1; 3	7	1	2; 4	**2; 0**	2	1	1
NL-63-related bat CoV			BtKYNL63-9b	NC_048216	**0**	1	**0**	**0**	2	4*	2	3*	2*; 3*	3; 2; 3	3; 5	4	1	**0; 2**	**0; 0**	2	1	1
Nyctalus velutinus alphaCoV			SC-2013	NC_028833	**0**	1	**0**	1	4	5*	1	5*	2*; 3*	4; 3; 2	2; 4	5	1	1; 3	**0; 0**	**0**	1	2
Pipistrellus khulii coronavirus			P.kuhlii/Italy/3398-19/2015	NC_046964	**0**	**0**	**0**	1	5	7*	2	6*	2*; 3*	6; 5; 1	2; 4	9	1	3; 5	**0; 0**	1	1	1
Porcine epidemic diarrhea virus			CV777	NC_003436	**0**	**0**	1	1	1	3*	4*	2*	1*; 4*	3; 0; 5	3*; 4*	4	1	**0; 2**	1; 1	**0**	1	3*
Rhinolophus ferrumequinum alhaCoV			HuB-2013	NC_028814	**0**	**0**	1	1	2	3	3	3*	4*; 2*	5; 3; 4	6; 7	5	2	2; 4	3; 1	**0**	2	4
Rhinophilus bat coronavirus HKU2			HKU2/HK/46/2006	EF203065	**0**	1	1	**0**	2	4*	3	3*	2; 3	3; 2; 3	3; 3	5	1	**0; 2**	**0; 0**	1	1	2
Scotophilus bat coronavirus			512/2005	NC_009657	**0**	1	1	1	2	4*	2	3*	2; 2	4; 1; 5	3; 5	5	1	**0; 2**	**0; 0**	1	2	1
Sorex araneus coronavirus			Shrew-CoV/Tibet2014	NC_046955	**0**	1	**0**	**0**	3	4*	3	3	2*; 4*	4; 3; 3	4; 4	4	1	1; 3	**0; 0**	1	2	2
Suncus murinus coronavirus			Xingguo-101	NC_035191	**0**	**0**	2	1	5*	7*	4	6*	2; 1	3*; 5*,7*	5; 3	8	1	**2; 0**	**2; 0**	**0**	**0**	**3**

Table S2. Complementarity of primers with *Betacoronavirus* sequences. The primers that did not bind to the template in *in silico* analyses are underlined (*the mismatches were present within the 3 bp of 3′end of primer). Primer sets where at least one primer had no mismatch with the template sequence are in **bold**.

Species	Subspecies	Isolate	Accession No.	Hu-F	Hu-R	Chu11-F1	Chu11-R1	Chu06-R1	Chu06-R2	Poon-F	Poon-R	DeSouzaLuna-F1+2	DeSouzaLuna-R1+2+3	DeSouzaLuna-F3+4	DeSouzaLuna-R4	Holbrook-F1	Holbrook-R1+2	Holbrook-F2+3	Xiu-F1	Xiu-R	Xiu-F2
Betacoronavirus 1	Human CoV OC43	ATCC VR-759	AY585228	**0**	**0**	**0**	**0**	4	7*	1	4	**0; 3**	1; 4; 5	**0; 2**	6	1	**2; 0**	**0; 2***	**0**	1	**0**
	Bovine coronavirus	Mebus	BCU00735	**0**	**0**	**0**	**0**	4	7*	1	4	**0; 3**	1; 4; 5	**0; 2**	6	1	**2; 0**	**0; 2***	**0**	1	**0**
	PHEV	VW572	NC_007732	**0**	**0**	1	1	4	7*	2	4	**0; 3**	1; 4; 5	1; 2	5	1	**2; 0**	**0; 2***	1	2	1
	Equine coronavirus	NC99	NC_010327	**0**	1	**0**	**0**	4	7*	1	4	**0; 3**	1; 4; 5	**0; 2**	6	1	**2; 0**	**0; 2***	**0**	2	**0**
China Rattus coronavirus HKU24		HKU24-R05005I	NC_026011	**0**	**0**	**0**	1	4	7*	2*	4	1; 3	1; 4; 5	3*, 5*	7	1	3*; 1	**2; 0**	1	2	1
Eidolon bat coronavirus C704		Kenya/KY24/2006	HQ728482	1	**0**	**0**	1	4	7*	**0**	4	2; 2	2; 3; 7	3; 5	7	1	**0; 0**	**2; 0**	1	1	2
Hedgehog coronavirus 1		ErinaceusCoV/2012-174/GER/2012	NC_039207	**0**	1	**0**	1	2	4*	2	3*	1*; 4*	4; 1; 5	1; 3	5	1	**0; 2***	**0; 2**	1	1	2
Human coronavirus HKU1		HKU1	NC_006577	**0**	**0**	**0**	1	4	7*	2	4	**0; 3**	1; 4; 5	1; 1	6	1	**0; 2***	**2; 0**	1	**0**	1
MERS-related coronavirus		camel/UAE_B21_2015	MF598613	**0**	**0**	**0**	**0**	3	3*	2	3	1*; 4*	3; 2; 4	1; 3	5	4	**0; 0**	**0; 2**	1	2	2
		HCoV-EMC/2012	NC_019843	**0**	**0**	**0**	**0**	3	3*	2	3	1*; 4*	3; 2; 4	1; 3	5	1	**0; 2***	**0; 2**	1	3	2
Murine coronavirus	Rat coronavirus	Rat CoV Parker	FJ938068	**0**	**0**	**0**	1	4	7*	**0**	4	1; 2	1; 4; 5	1; 3	5	1	**2; 0**	**0; 2***	1	1	1
	MHV	JHM	NC_006852	**0**	**0**	**0**	1	5	8*	1	5	2; 2	2; 5; 6	3; 3	6	1	3*; 4*	**0; 2***	1	1	2
Myodes coronavirus 2JL14		RtMruf-CoV-2/JL2014	NC_046954	1	**0**	1	1	4	7*	3	4	1; 2	1; 4; 5	2; 2	6	1	**0; 2***	**2; 0**	2	2	2
Pipistrellus bat coronavirus HKU5		HKU5-1 LMH03f	NC_009020	**0**	1	1	**0**	3	5*	3	4*	2*; 3*	4; 3; 3	4; 4	6	1	**0; 2***	1; 3	2	1	2
Rousettus bat coronavirus HKU9		HKU9-1 BF_005I	NC_009021	1	**0**	**0**	1	4	7*	1	4	2; 1	2; 3; 6	4; 4	7	1	**0; 2***	3; 1	2	2	**0**
SARS-related CoV	human CoV	Urbani	AY278741	**0**	1	1	**0**	2	2	3	3*	**0; 3**	**3; 0; 5**	2; 1	4	1	1; 3	**0; 0**	2	1	2
	palm civet CoV	SZ3/2003	AY304486	**0**	1	1	**0**	2	2	3	3*	**0; 3**	**3; 0; 5**	2; 1	4	1	1; 3	**0; 0**	2	1	2
	chinese ferret-badger CoV	CFB/SZ/94/03	AY545919	**0**	**0**	1	**0**	2	2	3	3*	**0; 3**	**3; 0; 5**	2; 1	4	1	1; 3	**0; 0**	2	1	2
	SARS-CoV-2	human/IT/32898/2020	MT483878	**0**	**0**	**0**	1	4	4	2	5*	**0; 3**	5; 3; 4	1; 1	5	1	3; 5	**0; 0**	1	**0**	1
	Rhinopholus bat CoV	Rp3/2004	NC_009693	**0**	1	1	**0**	2	2	3	3*	**0; 3**	**3; 0; 5**	2; 1	5	1	1; 3	**0; 2***	2	1	2
		HKU3-1	NC_009694	**0**	**0**	1	1	3	3	3	4*	3*; 3*	4; 1; 4	5; 4	5	1	2; 4	**0; 2***	4	**0**	4
		Rf1/2004	NC_009695	**0**	**0**	1	1	2	2	3	3*	2*; 3*	**3; 0; 5**	4; 3	4	1	1; 3	**0; 2***	4	**0**	4
		Rm1/2005	NC_009696	**0**	1	1	1	2	2	1	3*	1; 2	**3; 0; 5**	2; 3	4	1	1; 3	**0; 2***	2	1	2
Tylonycteris bat coronavirus HKU4		HKU4-1 BO4f	NC_009019	**0**	1	**0**	**0**	3	4*	2	3	2*; 4*	6; 4; 3	3; 5	4	1	**0; 2***	2; 4	1	**0**	1
Bat Hp-betacoronavirus		Zhejiang2013	NC_025217	1	**0**	**0**	**0**	3	4*	1	3	1; 3	4; 3; 3	1; 3	6	1	**0; 2***	1; 3	**0**	3	1

Table S3. Complementarity of primers with *Gammacoronavirus* and *Deltacoronavirus* sequences. The primers that did not bind to the template in *in silico* analyses are underlined (*the mismatches were present within the 3 bp of 3′end of primer). Primer sets where at least one primer had no mismatch with the template sequence are in **bold**.

Genus	Species	Isolate	Accession No.	Hu-F	Hu-R	Chu11-F1	Chu11-R1	Chu06-R1	Chu06-R2	Poon-F	Poon-R	DeSouzaLuna-F1+2	DeSouzaLuna-R1+2+3	DeSouzaLuna-F3+4	DeSouzaLuna-R4	Holbrook-F1	Holbrook-R1+2	Holbrook-F2+3	Xiu-F1	Xiu-R	Hu-F2
δ-CoV	*Coronavirus HKU15*		LC216915	**0**	**0**	**0**	**0**	5*	9*	2	6*	3*; 5*	5*; 4*; 4*	4; 6	8*	1	**0; 2**	**2*; 0**	1	**0**	1
	Bulbul coronavirus HKU11-934		NC_011547	**0**	1	1	1	2*	6*	3	3*	3*; 5*	4; 2; 5	5; 5	4	1	**0; 2**	**2*; 0**	**0**	1	2
	Thrush coronavirus HKU12-600		NC_011549	**0**	**0**	**0**	1	5*	8*	2	6*	3*; 4*	4; 3; 3	4; 6	7	1	**0; 2**	**2*; 0**	**0**	1	1
	Munia coronavirus HKU13-3514		NC_011550	**0**	**0**	2	**0**	2*	6*	4	3*	4*; 5*	4; 2; 5	6; 6	4	1	**0; 2**	1*; 1*	**0**	1	3
	White-eye coronavirus HKU16		NC_016991	**0**	**0**	**0**	1	4*	7*	2	4*	3*; 5*	5*; 2*; 5*	4; 6	7*	1	**0; 2**	2*; 1*	**0**	1	1
	Night-heron coronavirus HKU19		NC_016994	**0**	1	**0**	**0**	3*	5*	3	4*	6*; 7*	2; 1; 4	7; 9	4	**0**	**0; 2**	**2*; 0**	1	1	2
	Wigeon coronavirus HKU20		NC_016995	**0**	1	**0**	**0**	4*	9*	2	5*	3; 5	6*; 4*; 6*	5; 5	8*	1	1; 3	**2*; 0**	1	**0**	1
	Common moorhen coronavirus HKU21		NC_016996	**0**	1	**0**	1	5*	9*	1	6*	3; 6	7*; 5*; 5*	3; 5	8*	1	1; 3	1*; 1*	**0**	2	**0**
	Coronavirus HKU15		NC_039208	**0**	**0**	**0**	**0**	5*	9*	2	5*	3*; 5*	5*; 4*; 4*	4; 6	8*	1	**0; 2**	**2*; 0**	1	**0**	1
γ-CoV	*Turkey coronavirus*	TCoV-ATCC	EU022526	**0**	**0**	**0**	**0**	6*	6	2	7*	1; 3	5; 4; 1	1; 3	6	**0**	2; 4	**0; 0**	1	1	1
	Avian coronavirus	Baudette	NC_001451	**0**	1	**0**	**0**	6*	7*	1	7*	**0; 3**	5; 4; 1	0; 2	6	1	2; 4	**0; 0**	1	2	**0**
	Beluga whale coronavirus SW1		NC_010646	1	2*	**0**	**0**	8*	11*	1	8*	3; 1	7*; 4; 5	3; 5	8	**0**	3; 1	**0; 0**	2	2	2
	Goose coronavirus	Cambridge_Bay_2017	NC_046965	**0**	1	1	1	4*	5*	2	4*	2; 2	4; 3; 2	3; 3	5	1	1; 3	1*; 1*	1	2	3
	Avian coronavirus 9203	IBV Ind-TN92-03	NC_048213	**0**	**0**	1	**0**	6*	6	2	7*	**0; 3**	5; 4; 1	1; 2	6	1	2; 4	**0; 0**	1	1	1
	Duck coronavirus 2714	DK/GD/27/2014	NC_048214	**0**	1	**0**	**0**	5*	6*	2	5*	**0; 3**	5; 4; 3	1; 1	6	1	2; 4	**0; 0**	**0**	**0**	1

Table S4. Field samples and viral strains used to test the inclusivity of the novel assay developed in this study.

Genus	virus	host	matrix tested	Year	Collection strategy	strain
**α-CoV**	Alphacoronavirus 1_Feline Coronavirus	Cat	viral strain (cell culture)			WSU 79-1683 (ATCC VR-989)
	Alphacoronavirus 1_Canine Coronavirus	Dog	viral strain (cell culture)			Jan-71 (ATCC VR-809)
	Alphacoronavirus 1_Ferret Coronavirus	Ferret	feces	2021	Dry	IZSVe21RS42-F
	Alphacoronavirus 1_Ferret Coronavirus	Ferret	intestine	2021	Dry	IZSVe21RS42-18
	Alphacoronavirus 1_TGE	Pig	viral strain (cell culture)			Virulent Purdue
	Miniopterus coronavirus 1	*Miniopterus schreibersii*	oral swab	2020	Lysis buffer	IZSVe21RS156-83
	PEDV	Pig	feces	2018	Dry	18DIA90174-11
	PEDV	Pig	feces	2018	Dry	18DIA90174-10
	Unclassified pipistrelle αCoV	*Pipistrellus kuhlii*	feces	2018	Viral transport medium	IZSVe19RS495-11
	Unclassified myotis αCoV	*Myotis myotis*	feces	2016	RNA later	IZSVE21RS474-25
	Unclassified myotis αCoV	*Myotis myotis*	feces	2016	RNA later	IZSVE21RS474-36
**β-CoV**	Betacoronavirus 1_PHEV	Pig	feces	2018	Dry	18DIA90174-2
	Betacoronavirus 1_PHEV	Pig	feces	2018	Dry	18DIA90178-5
	Betacoronavirus 1_PHEV	Pig	rectal swab	2021	RNA later	IZSVe21RS1031-16
	Betacoronavirus 1_Bovine Coronavirus	cow	viral strain (cell culture)			Mebus
	Betacoronavirus 1_Bovine Coronavirus	*Tragelaphus spekii*	feces	2019	Dry	IZSLER32286-1/2019
	Betacoronavirus 1_Bovine Coronavirus	*Tragelaphus spekii*	feces	2019	Dry	IZSLER32286-2/2019
	Murine Coronavirus	*Mus musculus*	feces	2021	Dry	IZSVe21RS42-T
	Betacoronavirus Erinaceus	*Erinaceus europaeum*	intestine	2021	Dry	IZSVe21RS156-83
	Unclassified Eptesicus βCoV	*Eptesicus serotinus*	feces	2011	Dry	ITA26_384_2012
	Unclassified Eidolon βCoV	*Eidolon helvum*	feces	2013	Dry	13RS452-71
	Unclassified Eidolon βCoV	*Eidolon helvum*	feces	2013	Dry	13RS362-59
	SARS-CoV-2	Dog	oral swab	2020	Dry	20DIA30037-1/2020
	SARS-CoV-2	Human	viral strain (cell culture)			20VIR1935
	SARS-CoV-2	Cat	nasal swab	2020	Dry	VE-IZSVe-20DIA30040-2/2020
**γ-CoV**	IBV	Chicken	viral strain (egg culture)			IZSVe21RS156-64
	duck coronavirus	*Anas platyrhynchos*	feces	2018	Dry	IZSLER138948-2/2018
	duck coronavirus	*Anas platyrhynchos*	feces	2019	Dry	IZSLER288952-1/2019
**δ-CoV**	quail coronavirus	*Coturnix japonica*	intestine	2019	Dry	IZSLER335273/2019
	quail coronavirus	*Coturnix japonica*	intestine	2019	Dry	IZSLER160792/2019
